# Open-source, vendor-independent, automated multi-beat tissue Doppler echocardiography analysis

**DOI:** 10.1007/s10554-017-1092-4

**Published:** 2017-02-20

**Authors:** Niti M. Dhutia, Massoud Zolgharni, Michael Mielewczik, Madalina Negoita, Stefania Sacchi, Karikaran Manoharan, Darrel P. Francis, Graham D. Cole

**Affiliations:** National Heart and Lung Institute, Imperial College London, Hammersmith Hospital Campus, Du Cane Road, London, W12 0NN UK

**Keywords:** Tissue Doppler, Echocardiography, Automated, Vendor-independent measurements, Myocardial velocities

## Abstract

Current guidelines for measuring cardiac function by tissue Doppler recommend using multiple beats, but this has a time cost for human operators. We present an open-source, vendor-independent, drag-and-drop software capable of automating the measurement process. A database of ~8000 tissue Doppler beats (48 patients) from the septal and lateral annuli were analyzed by three expert echocardiographers. We developed an intensity- and gradient-based automated algorithm to measure tissue Doppler velocities. We tested its performance against manual measurements from the expert human operators. Our algorithm showed strong agreement with expert human operators. Performance was indistinguishable from a human operator: for algorithm, mean difference and SDD from the mean of human operators’ estimates 0.48 ± 1.12 cm/s (R^2^ = 0.82); for the humans individually this was 0.43 ± 1.11 cm/s (R^2^ = 0.84), −0.88 ± 1.12 cm/s (R^2^ = 0.84) and 0.41 ± 1.30 cm/s (R^2^ = 0.78). Agreement between operators and the automated algorithm was preserved when measuring at either the edge or middle of the trace. The algorithm was 10-fold quicker than manual measurements (p < 0.001). This open-source, vendor-independent, drag-and-drop software can make peak velocity measurements from pulsed wave tissue Doppler traces as accurately as human experts. This automation permits rapid, bias-resistant multi-beat analysis from spectral tissue Doppler images.

**Electronic supplementary material:**

The online version of this article (doi:10.1007/s10554-017-1092-4) contains supplementary material, which is available to authorized users.

## Introduction

Tissue doppler imaging (TDI) is an echocardiographic technique that can assess left ventricular systolic and diastolic function [1–4]. It has diagnostic and prognostic utility across a wide range of scenarios including heart failure, cardiomyopathy and valvular disease [5–10]. Strain imaging is also useful for evaluation of myocardial ischemia–reperfusion [11].

Current guidelines suggest measuring and averaging at least three consecutive beats for tissue Doppler velocity measurements [1–3]. Although this may improve the precision of the measurement, asking humans to make multiple manual measurements from tissue Doppler traces is time-consuming and disruptive to workflow.

If an automated system could rapidly make measurements from tissue Doppler traces without disrupting the workflow, experts performing the scan could spend more time acquiring beats, improving precision and efficiency of echocardiography studies without consuming more time.

We have previously found that peak velocity measurements made at the middle of the Doppler envelope agree with different modalities better than measurements made at the outer edge of the Doppler envelope [12]. However, whether measuring from the middle or edge of the Doppler envelope is the more reproducible strategy remains controversial [13]. Ideally, an algorithm should be able to make measurements from both the middle and the outer edge of the tissue Doppler trace to allow a like-for-like comparison between algorithm and human experts.

Although algorithms exist for automated quantification of some aspects of cardiac function [14–16], there are no algorithms available ready for use by clinicians on systems from all vendors and with their internal workings exposed for others to verify and improve. We therefore developed, tested and evaluated a vendor-independent solution to make reproducible, bias-resistant, multi-beat tissue Doppler measurements.

## Methods

### Subjects

Pulsed-wave tissue Doppler traces were acquired from 48 patients (30 male). Patients had a mean age of 64 ± 11 years. Clinical characteristics of the patients are shown in Table 1. The patients investigated were recruited from patients who had undergone echocardiography with Imperial College Healthcare NHS Trust. Only patients in sinus rhythm were included. No other exclusion criteria were applied. The study was approved by the local ethics committee and written informed consent was obtained.

**Table 1:** Clinical characteristics of patients

Diagnosis	Number of patients n (%)
Hypertension	31 (65)
Coronary disease	18 (38)
Valvular disease	11 (23)
Impaired LV function	9 (19)
Diabetes	4 (8)
Thyroid	3 (6)
Previous CABG	2 (4)
Previous cardiac arrest	2 (4)
Liver disease	2 (4)
Chronic renal disease	1 (2)
Previous stroke	1 (2)
Pacemaker	1 (2)

### Data acquisition

Each patient underwent standard tissue Doppler assessment of left ventricular function by an experienced echocardiographer. The operator was advised to optimize the images as would normally be done in clinical practice. For all acquisitions, the sample volume size was 5 mm with a sweep speed of 75 mm/s. Traces from both the septal and lateral annuli were acquired for 30 s each. This entire process was conducted three times, with the probe removed from the chest and then placed back on the chest optimally between each recording. A total of six 30-second recordings (three at the septal annulus and three at the lateral annulus) of tissue Doppler data were acquired for each patient.

Images were acquired using a standard video capture device, live from the echocardiography machine’s external display output. In our study, we used a Philips iE33 ultrasound machine (Guildford, UK) with a VGA output, and the VGA2USB Pro (Epiphan Systems, Canada). The system works equally well with the GE Vivid i (with the same VGA video capture device) and with the DVI output of the Philips iE33, which requires a DVI capture card such as the DVI2USB (Epiphan Systems, Canada). The captured snapshots were acquired at a resolution of 900 × 1300 pixels and reconstructed into a continuous strip of Doppler data using an in-house developed Matlab program.

**Fig. 1:**
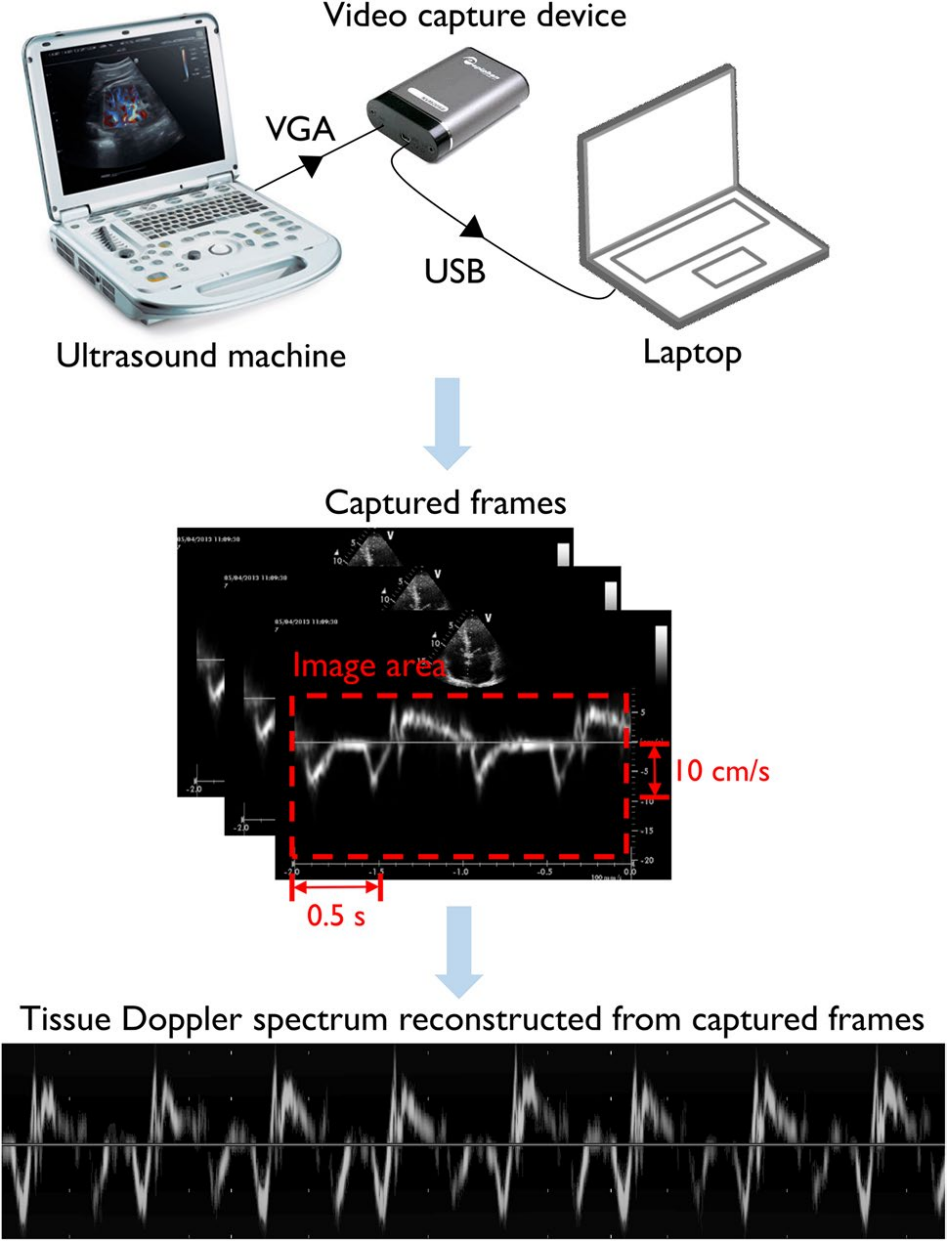
Acquisition procedure. Schematic showing the process of acquisition using a video capture device connected to any ultrasound machine with a VGA output port (*top* panel). Image area and scale are extracted from acquired snapshots (*middle* panel) and reconstructed to obtain an extended tissue Doppler spectrum (*bottom* panel)

Each echocardiography system has a small number of spatial templates for displaying items on the screen such as the trace itself, the 2D B-mode preview, text and annotations. The algorithm automatically detects which template is active and crops the overall image down to just the Doppler trace itself. The horizontal zero-velocity axis was automatically detected by averaging across every horizontal row in the image and selecting the one with the highest mean intensity. Template matching based optical character recognition (Computer Vision System Toolbox, Matlab, Mathworks) was used to locate and read out the scaling of the velocity (cm/s) and time axes (seconds). The steps involved in the image acquisition and reconstruction procedure are summarized in Fig. 1. We report further details on reconstruction of the Doppler strips elsewhere [17].

### Development of an automated image analysis algorithm

**Fig. 2:**
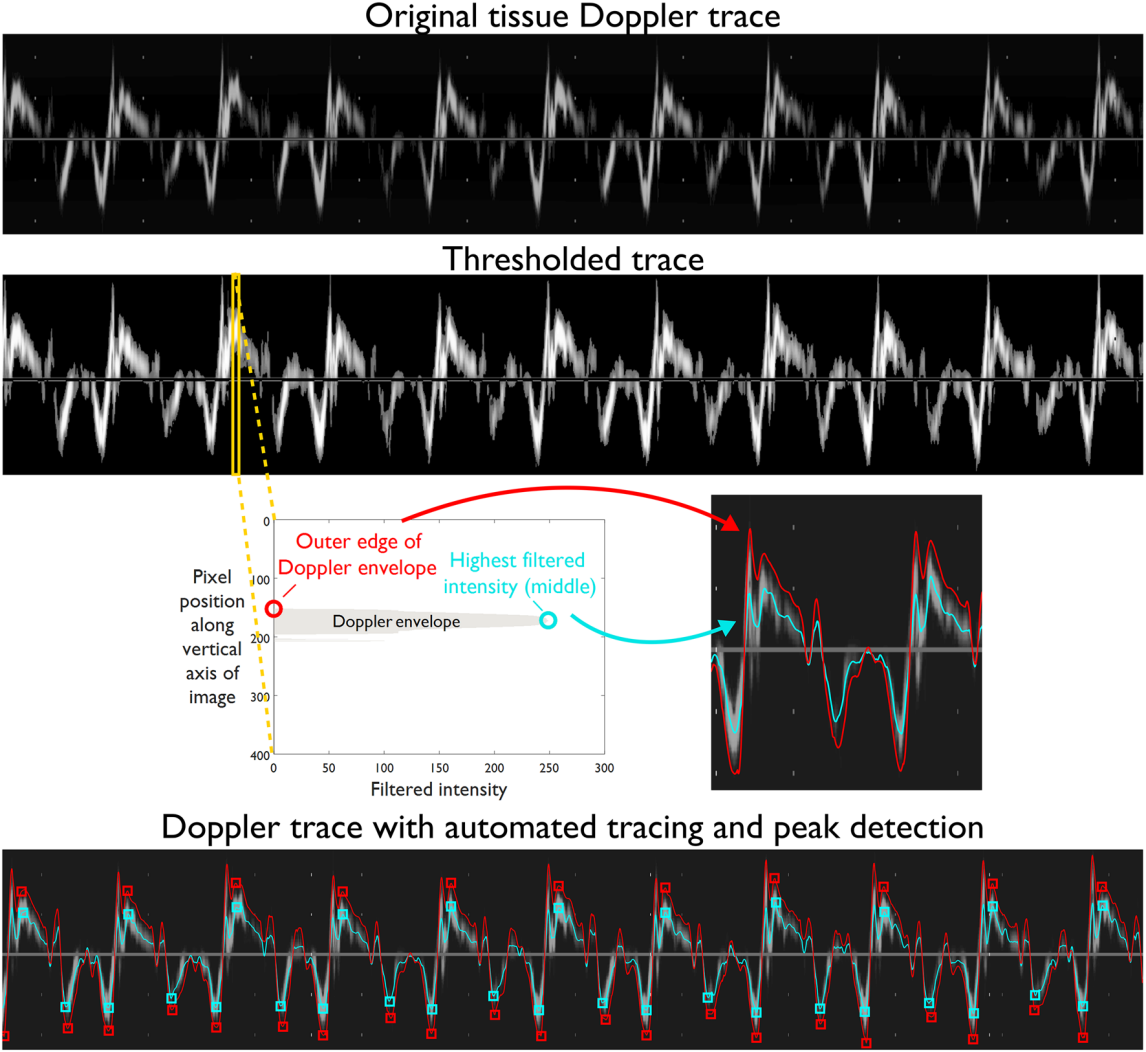
Processing steps for automated tracing and extracting peak velocities. The reconstructed tissue Doppler trace (*top* panel) is thresholded to separate foreground pixels (trace) from background pixels (*second* panel). Each column of this image (*yellow bar*) is analyzed to find the location of the highest filtered intensity (*third* panel schematic—*left*) which is defined as the middle of the envelope (*blue circle*), and largest drop in pixel intensity which is defined as the outer edge of the envelope (*red circle*). The outer edge (*red*) and middle (*blue*) traces are shown overlaid on the Doppler trace after analyzing each vertical column (*third* panel—*right*). The *bottom* panel shows the extended Doppler spectrum with automated outer (*red*) and middle (*blue*) traces with peak systolic (s′) and diastolic (e′ and a′) velocities (*red* and *blue squares*)

Figure 2 shows the schematic of processing steps involved in the automated algorithm’s analysis. An Otsu threshold filter [18] was applied to the tissue Doppler images, automatically selecting a cut-off threshold based on the underlying pixel intensities to separate the background pixels from the trace in the foreground. Additionally, the zero axis line was replaced by zero intensity pixels, i.e., background, to avoid interference with further analysis of the image and subsequent velocity curves.

Each vertical column in the image was filtered using a low-pass Butterworth filter [19] for noise reduction. This filter helps to eliminate any background noise not removed by the thresholding. The algorithm then locates the pixel with the highest filtered intensity which in this paper we refer to as the “middle” of the envelope. Statistically this value corresponds to the mode of the filtered velocities at that time-point. If the distribution of velocities near the mode is approximately symmetrical, it will also be the mean velocity [12]. The outer edge of the tissue Doppler envelope was identified for each vertical column by locating the location of the sharpest drop in filtered pixel intensities (i.e., largest gradient in amplitude), moving outwards from the middle pixel. The resulting velocity curves (middle and outer envelope) were smoothed using a simplified least-squares filter (Savitzky–Golay) [20]. These parameters were found to achieve an acceptable trade-off between the strength of the filter in noise removal and retention of useful information. The downloadable software (Online Resource 1) includes a module to allow users to modify these parameters if desired.

Based on autocorrelation, commonly used in ECG analysis [21], an approximate length of a cardiac cycle was estimated. For each of the velocity traces (middle and outer edge) the algorithm then determined the systolic and diastolic peak velocities (s′, e′, a′) for each beat. The previously estimated cardiac cycle length was used to restrict the minimum distance between peaks during peak detection to prevent noise spikes from being falsely detected as peak velocities.

The results were exported to Excel files and the estimated peak points and velocity curves overlaid and superimposed on the acquired Doppler images (Online Resource 2).

The algorithm is freely available to download in executable format (Online Resource 1). Input images can be either standard DICOM images from any manufacturer or long strips acquired using any video capture device.

### Evaluation of the automated algorithm

To evaluate the agreement of our algorithm with expert operators, we compared the peak velocity values as measured by our algorithm with manual measurements from three expert echocardiographers. Agreement amongst the three experts and between the consensus of the experts and the algorithm was evaluated. Manual and automated measurements were made using two different measurement conventions (middle and outer edge of the Doppler envelope), in order to compare the agreement under either convention. The time to measure peak velocity for each beat using manual and automated methods was also recorded.

We tested the performance of the algorithm in two different ways.

First, we calculated for each patient, the average of the automated measurements and the average of each human experts’ measurements made from all the beats at the septal annulus, and similarly at the lateral annulus.

Second, a custom script was written to identify individual beats which were measured by at least one human operator and the algorithm for a beat-to-beat analysis.

This analysis was undertaken separately for measurements made at the outer edge and middle of the trace.

In order to provide an additional comparative overview, a random cross-section of 10% of the dataset was re-presented to the same human experts to evaluate the intra-operator agreement, which was analysed separately for combinations of walls (septal and lateral), measurements (s′, e′ and a′) and measurement conventions (outer edge or middle of trace).

### Standard DICOM images from different vendors

Our main protocol uses video capture to obtain extended Doppler traces of up to 30 s. In current clinical practice, because measurements are manual, often only 2–3 beats are acquired, as a single screen capture. To test the performance of our algorithm when given such restricted data and to test vendor-independence, we ran the algorithm with 40 previously acquired, standard DICOM images obtained from scanners of four different vendors.

### Statistical analysis

We displayed the automated and human read data (and between different human operators) using Bland–Altman plots. We calculated the bias and 95% limits of agreement (±1.96 SD) between the automated and manual measurements [22]. This was undertaken for s′, e′ and a′ velocities, and for measurements made at both the lateral and septal walls. In general, Bland–Altman plots are not ideal for plotting large number of data points. In our analysis, in order to optimally plot over 7500 beats, we modified the Bland–Altman plots by introducing alpha-transparency (α = 0.1).

We calculated the standard deviation and R-squared value from linear regression analysis for both the automated and manual measurements. To avoid bias, each expert’s measurements were compared with the consensus of the experts excluding that individual expert.

The level of statistical significance was set at p < 0.05.

## Results

### Choice of beats to measure

97% of the beats measured by the automatic algorithm were measured by at least one human operator. 96% of the beats measured by at least one operator were also measured by the automated algorithm. In total, 7582/8101 (93%) of the beats that were measured by human or algorithm were measured by both human and algorithm. These were analyzed for beat-to-beat agreement.

### Agreement between experts and the automated algorithm

**Fig. 3:**
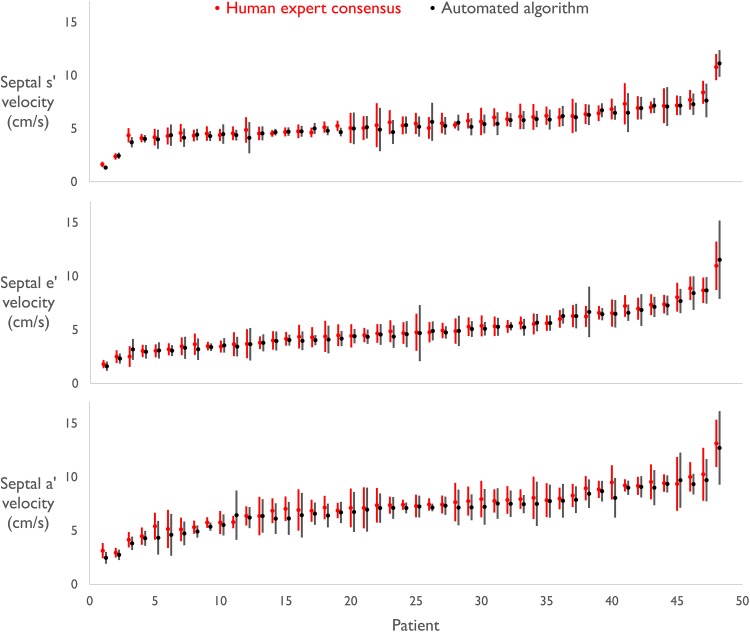
Average velocity estimates of experts versus automated algorithm. Average septal s′, e′ and a′ velocity estimates and standard deviations at the middle of the Doppler envelope across 90 s using the experts’ consensus (*red*) and the automated algorithm (*black*) for each patient. The markers represent average velocity across 3 × 30 s recordings (94 ± 32 beats per patient) and *error bars* represent standard deviation for those beats. The patients have been placed in ascending order of velocity

Figure 3 shows the average automated and human experts’ velocity measurements across 90 s at the septal annulus for each patient for systolic, early diastolic and late diastolic phases. The number of beats measured for each patient was 94 ± 32. Velocities ranged from 1.3 to 11.5 cm/s, 1.5 to 14.0 cm/s and 2.5 to 14.4 cm/s for septal s′, e′ and a′ peaks respectively. Average lateral annulus measurements for each patient are shown in Online Resource 3. At the lateral annulus velocities ranged from 1.6 to 13.5 cm/s, 1.8 to 13.2 cm/s and 1.8 to 16.2 cm/s for s′, e′ and a′ peaks.

**Fig. 4:**
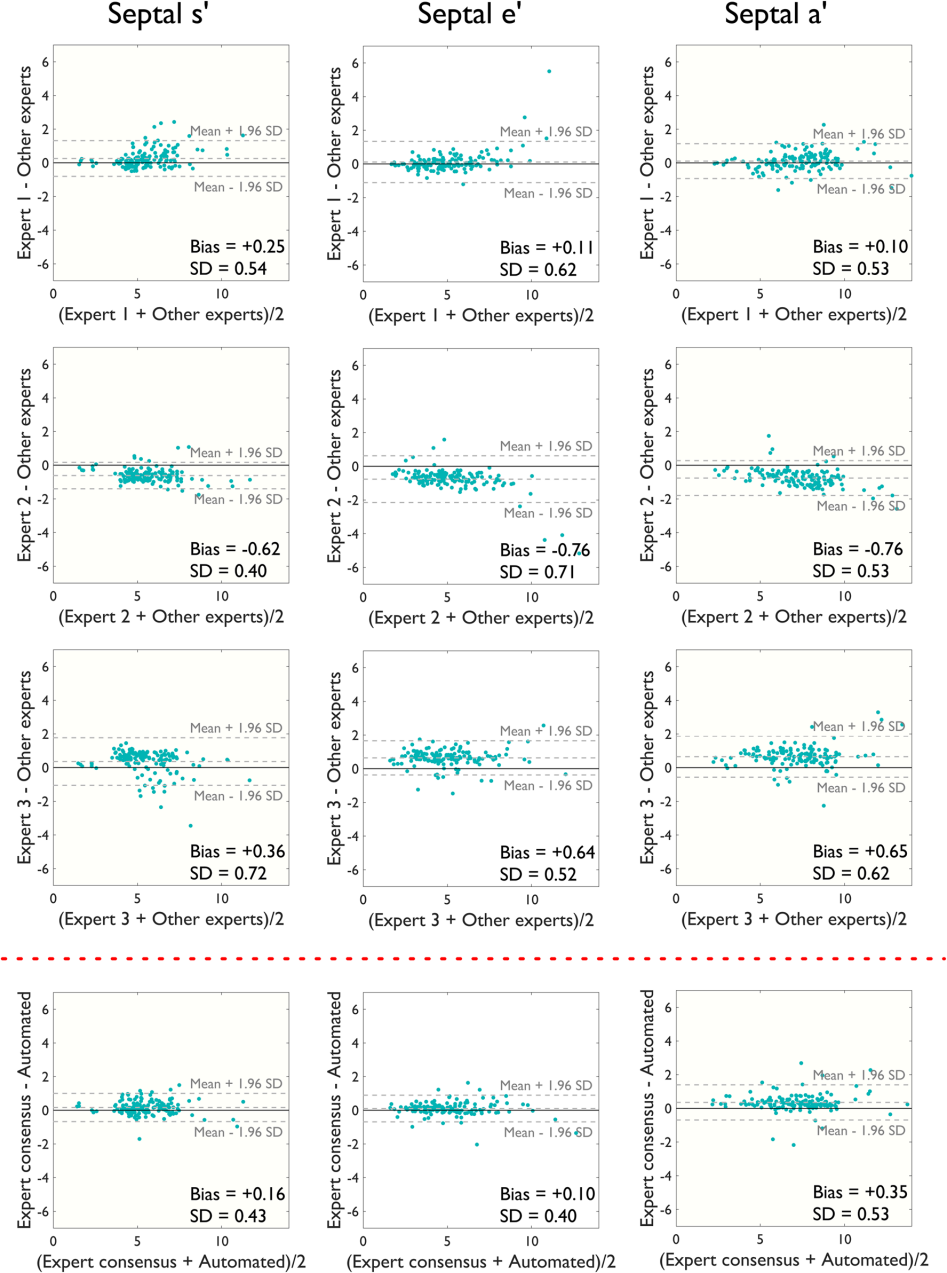
Bland–Altman plots for patient-by-patient analysis at the septal annulus. Bland–Altman plots comparing manual estimates from three experts and the automated algorithm’s estimates for septal peak s′, e′ and a′ velocities on a patient-by-patient basis

Figure 4 shows Bland Altman plots of the degree of disagreement between the experts, and also between algorithm and experts for the septal annulus. Each expert’s velocity measurements were compared with the consensus of the other two experts to avoid bias. Since the patients were approached without regard to whether they were likely to be good echo subjects, in some cases, image quality was poor which may have contributed to the wide dispersion in measurements between humans, and between algorithm and humans. To enable readers to judge the spectrum of image qualities, we show in Online Resource 2, a sample of ten of the images. They were selected to span the range of levels of disagreement from the first image with the greatest disagreement to the tenth image with the least disagreement.

**Fig. 5:**
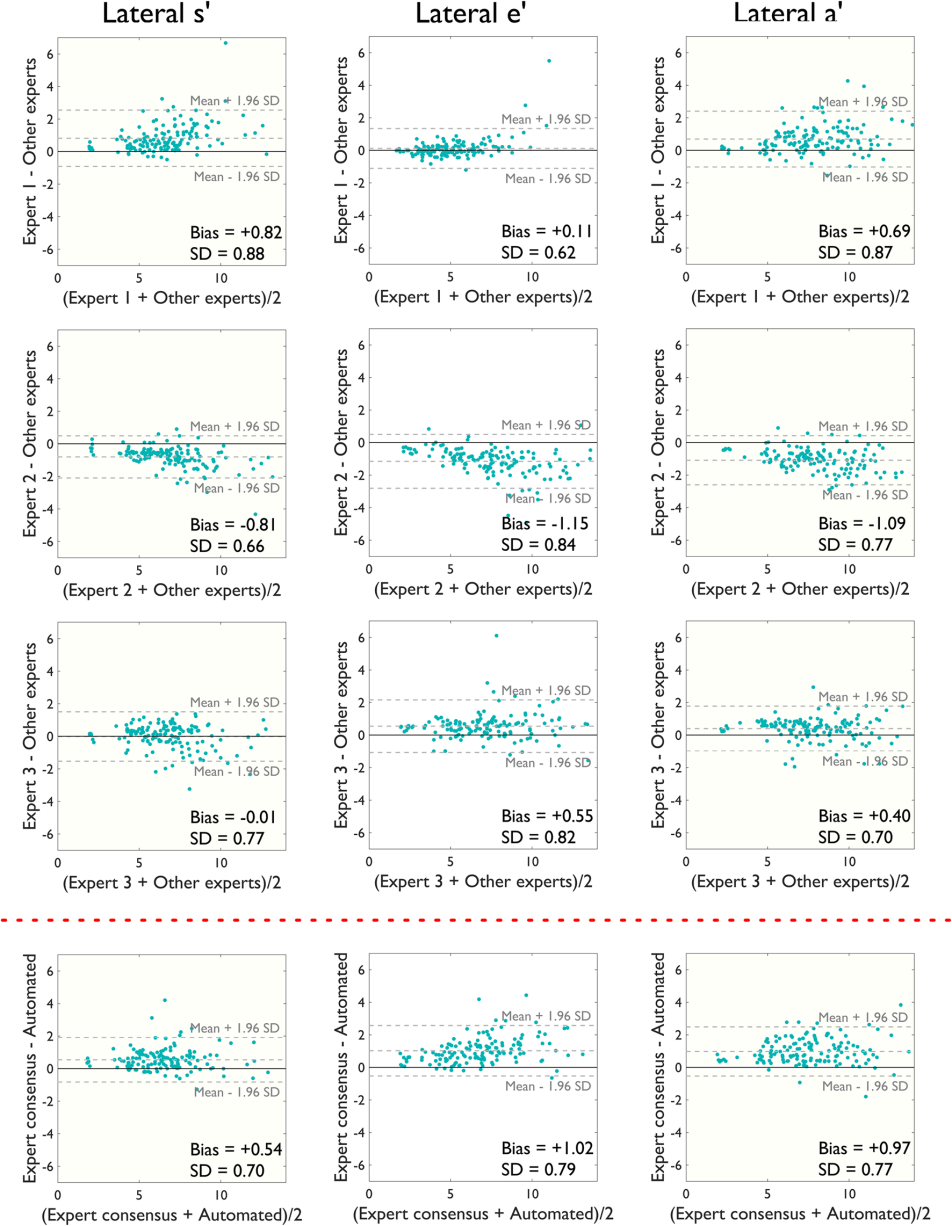
Bland–Altman plots for patient-by-patient analysis at the lateral annulus. Bland–Altman plots comparing manual estimates from three experts and the automated algorithm’s estimates for lateral peak s′, e′ and a′ velocities on a patient-by-patient basis

Figure 5 shows the corresponding data for the lateral annulus. There was greater disagreement for the lateral wall velocity measurements than the septal (mean difference of 0.54 ± 0.70 cm/s between algorithm and humans for septal s′ velocities at the lateral annulus vs. 0.16 ± 0.43 cm/s at the septal annulus). Bland–Altman biases and 95% limits of agreement are shown in Table 2 and R-squared values from linear regression in Table 3.

**Table 2:** Bland–Altman bias and 95% limits of agreement comparing measurements by experts with the automated algorithm

	Algorithm vs. expert consensus	Expert 1 vs. other experts	Expert 2 vs. other experts	Expert 3 vs. other experts	Expert 1 vs. algorithm	Expert 2 vs. algorithm	Expert 3 vs. algorithm
Septal annulus							
s′	0.16 ± 0.84	0.25 ± 1.06	−0.62 ± 0.78	0.36 ± 1.41	0.00 ± 0.84	0.57 ± 0.86	−0.08 ± 1.50
e′	0.10 ± 0.79	0.11 ± 1.22	−0.76 ± 1.39	0.64 ± 1.02	0.03 ± 1.12	0.60 ± 1.09	−0.33 ± 1.17
a′	0.35 ± 1.04	0.10 ± 1.03	−0.76 ± 1.03	0.65 ± 1.22	0.28 ± 1.10	0.84 ± 1.29	−0.09 ± 1.37
Lateral annulus							
s′	0.54 ± 1.37	0.82 ± 1.73	−0.81 ± 1.29	−0.01 ± 1.52	−0.01 ± 1.48	1.07 ± 1.76	0.55 ± 1.84
e′	1.02 ± 1.55	0.61 ± 1.34	−1.15 ± 1.65	0.55 ± 1.62	0.61 ± 1.54	1.79 ± 2.29	0.66 ± 1.66
a′	0.97 ± 1.51	0.69 ± 1.71	−1.09 ± 1.51	0.40 ± 1.38	0.52 ± 1.61	1.70 ± 2.07	0.72 ± 1.76

Bland–Altman bias and 95% limits of agreement comparing manual and automated peak tissue Doppler velocity (s′, e′ and a′) measurements at the septal and lateral annulus

**Table 3:** R-squared values from linear regression comparing measurements by experts with the automated algorithm

	Algorithm vs. expert consensus	Expert 1 vs. other experts	Expert 2 vs. other experts	Expert 3 vs. other experts	Expert 1 vs. algorithm	Expert 2 vs. algorithm	Expert 3 vs. algorithm
Septal annulus							
s′	0.93	0.90	0.94	0.83	0.93	0.93	0.80
e′	0.96	0.91	0.91	0.93	0.92	0.94	0.90
a′	0.93	0.94	0.95	0.91	0.92	0.92	0.88
Lateral annulus							
s′	0.90	0.85	0.93	0.88	0.87	0.85	0.83
e′	0.91	0.94	0.92	0.90	0.90	0.84	0.89
a′	0.90	0.89	0.93	0.92	0.88	0.85	0.87

R-squared values from linear regression comparing manual and automated peak tissue Doppler velocity (s′, e′ and a′) measurements for the septal and lateral annulus (p < 0.001 for all)

There was no significant difference in the performance of the software between different disease types.

### Algorithm performance with individual beat analysis

**Fig. 6:**
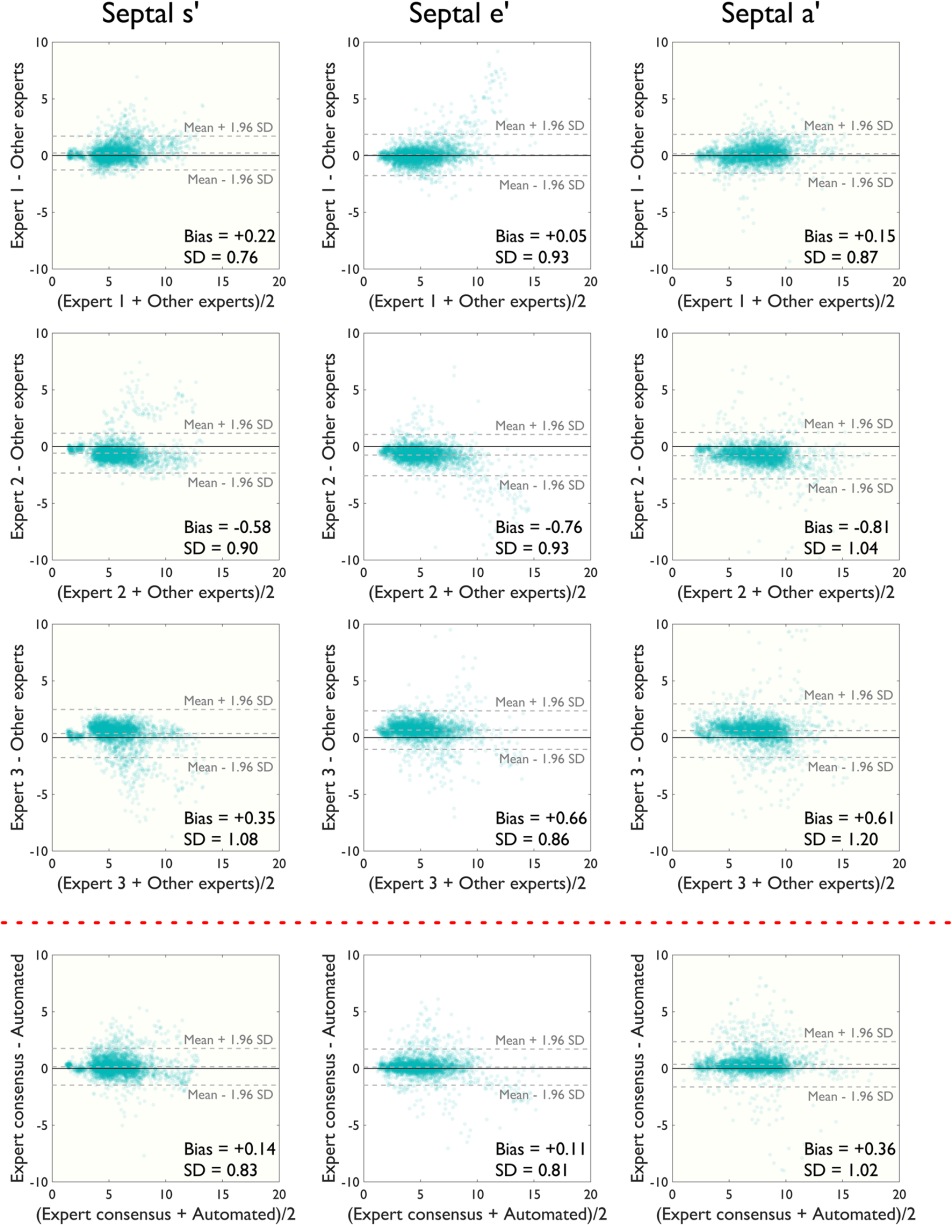
Modified Bland–Altman plots comparing manual estimates from three experts and the automated algorithm’s estimates for septal peak s′, e′ and a′ velocities on a beat-by-beat basis. An alpha-transparency of 0.1 has been applied to the *plotted markers*

**Fig. 7:**
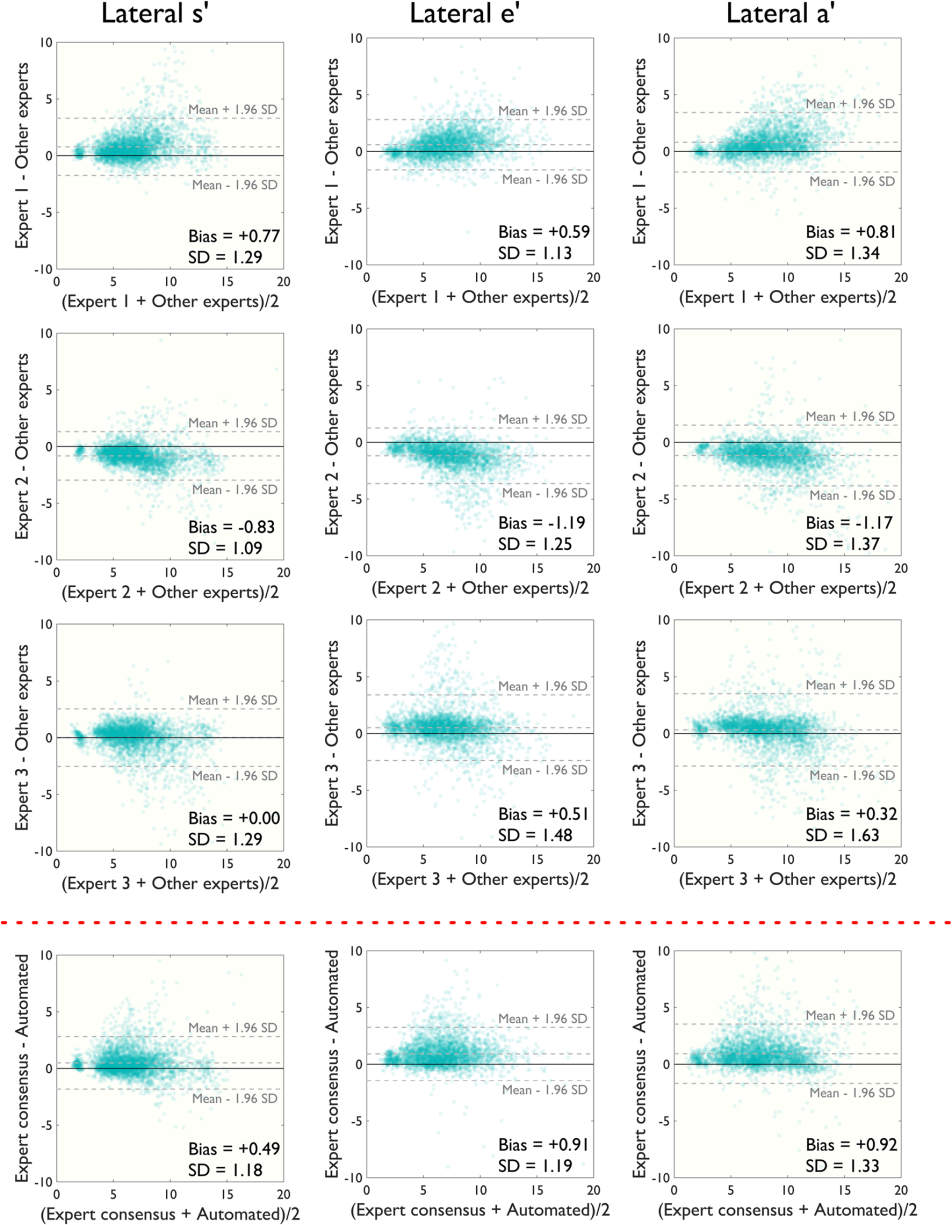
Bland–Altman plots for beat-by-beat analysis at lateral annulus. Modified Bland–Altman plots comparing manual estimates from three experts and the automated algorithm’s estimates for lateral peak s′, e′ and a′ velocities on a beat-by-beat basis. An alpha-transparency of 0.1 has been applied to the *plotted markers*

For individual beats (Figs. 6, 7), the automated algorithm agreed with the consensus of human operators as well as human operators agreed with each other: the beat-to-beat standard deviation of differences between the automated algorithm and consensus of expert estimates was 0.83 cm/s and between each expert’s estimates and consensus of experts excluding that individual expert was 0.76, 0.90 and 1.08 cm/s for septal s′ velocity measurements. The performance was similar for septal e′ (0.81 cm/s between algorithm and experts and 0.93, 0.93 and 0.86 cm/s between experts) and a′ (1.02 cm/s between algorithm and experts and 0.87, 1.04 and 1.02 cm/s between experts).

For the data which was re-presented to the same experts (10% of the dataset), the intra-operator standard deviation averaged 0.71 cm/s and the and inter-operator standard deviation averaged 0.37 cm/s. On average, 78% of the variance was intra-operator, compared with only 22% inter-operator.

### Agreement on the middle of the tissue Doppler envelope versus on the outer edge

Whilst example images from guidelines and teaching documents suggest measuring the outer edge of the envelope [1, 2, 23, 24], experimental data suggest that it is the middle of the trace that agrees best with other modalities [12]. It has been controversial which convention introduces more measurement error and/or reduces reproducibility [13].

**Fig. 8:**
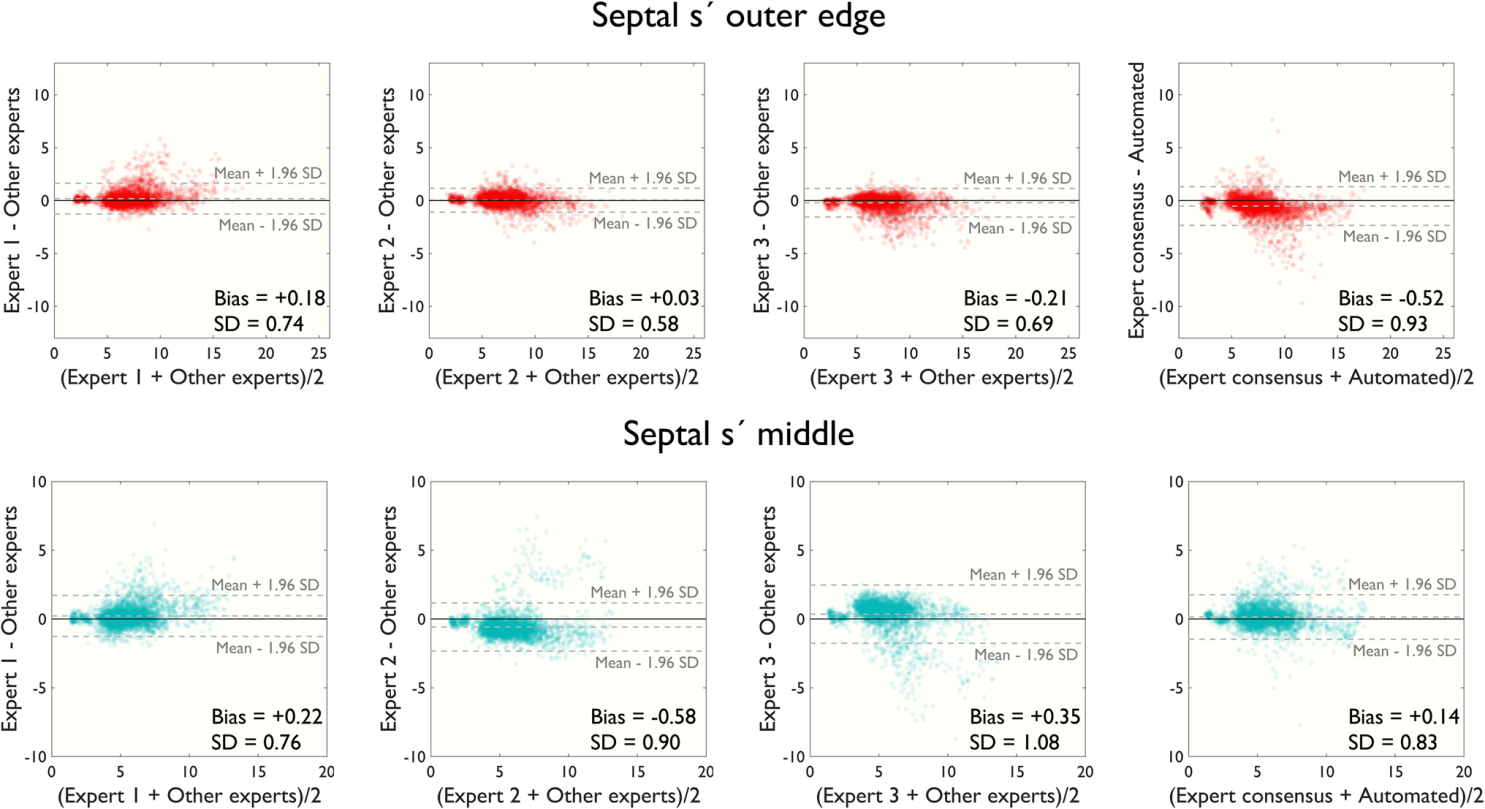
Outer edge versus middle of envelope. Modified Bland–Altman plots comparing two different measurement conventions for septal s′ measurements: outer edge (*top* panels) and middle (*bottom* panels) of the Doppler envelope. An alpha-transparency of 0.1 has been applied to the *plotted markers*

In the present study, we found that agreement was similar for the middle of the trace and the edge of the trace (Fig. 8). This was the case for both, agreement between humans and agreement between the algorithm and humans. Biases and 95% limits of agreement are shown in Table 4.

**Table 4:** Bland–Altman bias and 95% limits of agreement comparing measurements on the outer edge of the Doppler envelope with those at the middle of the Doppler envelope

	Algorithm vs. expert consensus	Expert 1 vs. other experts	Expert 2 vs. other experts	Expert 3 vs. other experts	Expert 1 vs. algorithm	Expert 2 vs. algorithm	Expert 3 vs. algorithm
Septal s′							
Middle	0.16 ± 0.84	0.25 ± 1.06	−0.62 ± 0.78	0.36 ± 1.41	0.00 ± 0.84	0.57 ± 0.86	−0.08 ± 1.50
Outer edge	−0.50 ± 1.29	0.16 ± 0.99	0.04 ± 0.48	−0.21 ± 0.91	−0.61 ± 1.61	−0.53 ± 1.30	−0.37 ± 1.27
Septal e′							
Middle	0.10 ± 0.79	0.11 ± 1.22	−0.76 ± 1.39	0.64 ± 1.02	0.03 ± 1.12	0.60 ± 1.09	−0.33 ± 1.17
Outer edge	−0.23 ± 1.19	−0.10 ± 1.16	0.10 ± 1.58	0.00 ± 1.46	−0.17 ± 1.47	−0.30 ± 1.46	−0.23 ± 1.62
Septal a′							
Middle	0.35 ± 1.04	0.10 ± 1.03	−0.76 ± 1.03	0.65 ± 1.22	0.28 ± 1.10	0.84 ± 1.29	−0.09 ± 1.37
Outer edge	−0.12 ± 1.32	−0.29 ± 1.27	0.23 ± 1.28	0.06 ± 1.80	0.07 ± 1.65	−0.27 ± 1.32	0.16 ± 1.91

Bland–Altman bias and 95% limits of agreement comparing manual and automated peak tissue Doppler velocity measurements at the septal annulus using different measurement conventions: middle of the Doppler envelope and outer edge of the Doppler envelope

### Feasibility of processing images from multiple vendors

To determine whether our algorithm works on ultrasound images acquired on more than one vendor’s equipment, we ran a previously acquired set of traces, obtained from scanners of four different vendors, each spanning a range of peak velocities. Images from the different vendors are shown in Online Resources 4 to 7, with annotations from the automated algorithm.

### Computation time

On average, the time required by the expert operators to make peak velocity measurements was reduced 10-fold by using the automated algorithm (p < 0.001). The average time taken for the automated peak measurements was 0.27 ± 0.05 s/beat. Our code is currently written in Matlab (Mathworks). If implemented in a low-level programming environment such as C++, the automated algorithm would further reduce the processing time required and could potentially perform the analysis in real time, with the result available immediately after acquisition of the image.

## Discussion

We present an open-source, vendor-independent automated algorithm to make peak velocity measurements from pulsed wave tissue Doppler traces. Current clinical practice guidelines suggest operators should make manual measurements from multiple beats on tissue Doppler traces. However, they also recognize that the analysis of these can be too time-consuming to carry out in the real world, and suggest a representative beat could be used instead [25]. Importantly, it is not the acquisition of multiple beats which is excessively time consuming, but the analysis. Our automated algorithm provides rapid and reliable measurements when given a small number of beats as is recommended in clinical practice. A human operator faced with acquiring and measuring from 2 to 3 beats could instead devote those ~30 s to acquiring a longer Doppler sequence from which this algorithm can make measurements with no additional time. Precision is improved [26] with no time cost.

The inter-observer variability in our study of ~8,000 beats was similar to that reported in the literature [10], and the automated algorithm’s performance is indistinguishable from this. Although an algorithm will always provide the same output when analyzing the same input image, this is not the case for humans who are subject to intra-operator variability and may make a different measurement when faced with the same trace. A subsample of data, when re-presented to the same experts, indicated that the majority of operator variance appears to be intra-operator rather than inter-operator. Due to this variation in manual measurements it is unlikely that the algorithm will agree better with the humans than they agree with themselves, and therefore the performance of the algorithm appears to be already very good. Nevertheless, we include both the executable file and our full source code so that anyone can inspect, test and improve upon them.

### Edge or middle, lateral or septal?

It is controversial whether to measure the middle or outer edge of a tissue Doppler trace [12, 13]. There was no significant difference in agreement between experts and the automated algorithm and agreement between experts for measurements made at the edge and middle of the Doppler trace. Our previous work indicated that measurements made using the middle of the Doppler envelope are most consistent with other modalities (M-mode and speckle tracking). The present study suggests that agreement between human observers, and between algorithm and observers, is similar for the middle line and the outer line conventions.

In contrast, measurements at the lateral annulus showed significantly more disagreement than those made at the septal annulus.

Examination of the Doppler traces suggests poorer quality images at the lateral annulus. We show examples (chosen by a systematic protocol) in Online Resource 8 for the lateral annulus and same patients’ septal annulus. We speculate this could be because the lateral annulus is more vulnerable to artefacts, out-of-plane movement and out-of-focus positioning.

### Open-source software

Open-source platforms allow dissemination of modern technological advances and can be used as a pathway to create improved algorithms that are both pragmatic and cost efficient. We provide our algorithm as a drag-and-drop program (Online Resource 1) for any reader to use on standard DICOM images from any vendor or time-extended acquisitions using the display output from the echocardiographic machine. We think it is important that colleagues can check its performance for themselves.

Readers may want to analyze already acquired Doppler traces. We therefore tested the functioning of the system on shorter 2–3 beat acquisitions common in clinical practice. It functions well on images from the four vendors we tested but we welcome broader testing, and, where necessary, improvement by readers.

### Study limitations

The performance of our algorithm was compared against the consensus of experts’ measurements. We believe this is a practical gold standard if we want to ensure that any deviations are not due to any changes in biological state from one beat to another or one time point to another. Such variations can be quite large compared to differences between patients, as shown by the standard deviation bars in Fig. 3, for example. (It should be remembered that the 95% ranges are twice the width of the error bars drawn.)

The patients were a convenience sample drawn from those attending a cardiology outpatient clinic. They therefore may not be representative of patients who enter trials with particular enrolment criteria or of inpatients or of the general population. However, since the algorithm does not rely on any specific features in the traces, there is no reason why it should not work for a wide range of subjects in any cardiovascular disease setting.

We only included patients in sinus rhythm, since the regular cycle length is part of our algorithm. A future evolution of the software will need to address the irregular cycle length of atrial fibrillation.

### Clinical implications

There are multiple sources of variability in Doppler measurements [27]. Our automated system can help in two ways. Firstly, it eliminates the variability that arises when different operators select different positions to make velocity measurements from the same images. Secondly, by allowing the analysis of multiple beats in the same time a human would take to measure a smaller number of beats, it can reduce the contribution of beat-to-beat variability. Similarly, the improved workflow might allow operators to remove the probe from the chest and replace it in another optimal position, reducing the contribution of probe placement.

An open-source, vendor independent algorithm also allows multicenter studies to be analyzed on a standardized platform.

An additional benefit may be as a tool for quality control. The use of an impartial non-human algorithm allows operators to evaluate themselves rapidly against an unbiased comparator.

It may also be possible to integrate our algorithm into other systems. We have previously developed an automated algorithm to automate the selection of the Doppler sample volume at the mitral valve annulus [28]. Mitral annular motion is known to be a good surrogate measure of overall longitudinal left ventricular contraction and relaxation [10]. A system automating the entire measurement process, including selection of the sample volume and making peak velocity measurements from the resulting Doppler trace, could be a useful tool. Focused echocardiography could benefit from this automated technology since it might allow non-specialists to obtain key velocity measurements and provide a rapid quantitative assessment of indices of left ventricular function [29].

## Conclusion

In this study we present an open-source, vendor-independent, drag-and-drop software to make measurements on tissue Doppler traces, downloadable from the Online Resources. The automated algorithm agreed with experts as well as they agreed with each other and reduced the time taken to acquire and measure a beat by 10-fold. Our algorithm could be useful in both research and clinical practice to help provide a rapid, reproducible and bias-resistant measurements of left ventricular function.

## Electronic supplementary material

Below is the link to the electronic supplementary material.

Supplementary material 1 (PDF 429 KB)

Supplementary material 2 (PNG 3382 KB)

Supplementary material 3 (PNG 1070 KB)

Supplementary material 4 (PNG 18324 KB)

Supplementary material 5 (PNG 10818 KB)

Supplementary material 6 (PNG 18054 KB)

Supplementary material 7 (PNG 15180 KB)

Supplementary material 8 (PNG 6353 KB)
