# Supplementary material for: Open-source, vendor-independent, automated multi-beat tissue Doppler echocardiography analysis
Source: Int J Cardiovasc Imaging. 2017 Feb 20;33(8):1135–48. doi: 10.1007/s10554-017-1092-4 (PMC5501914; doi:10.1007/s10554-017-1092-4)
Supplement: Supplementary file 1 — Supplementary material 1 (PDF 429 KB) [file 10554_2017_1092_MOESM1_ESM.pdf]

Imperial College London

Vendor-independent quantification of pulse-wave Doppler recordings

This document shows how to install and use the automated Doppler analysis software that we have provided with our paper, so that you can confirm, extend or refute what we have reported in our paper.

You will require:

(a) a computer running Microsoft Windows (this has been tested on Windows 7 and Windows 8)
(b) The file Automated_Tissue_Doppler.exe from the Automated_Doppler_analysis_software folder (online supplement S7), which is downloadable as an online supplement with this paper
(c) Some pulse-wave Doppler images you have exported from your echocardiography system as DICOM, BMP, PNG or JPEG freeze frames or extended video-captured files

Installing the tissue Doppler analysis program

1. Download and run the Automated_Tissue_Doppler.exe file from the Automated_Doppler_analysis_software folder from: https://onedrive.live.com/redir?resid=17610EBF453EF515!92166&authkey=!AGhimBJK0Ln0ENI&ithint=folder%2cpdf

2. Follow the on-screen instructions which take you through installation of the software and required components.

Using the program to automatically analyse a tissue Doppler image

1. Double-click on Automated_Tissue_Doppler.exe located in '..\Program Files\Automated Doppler\application'.

2. In the window that appears (Fig. 1), you can edit the filter parameters used during the analysis, or to use the default values, click OK.

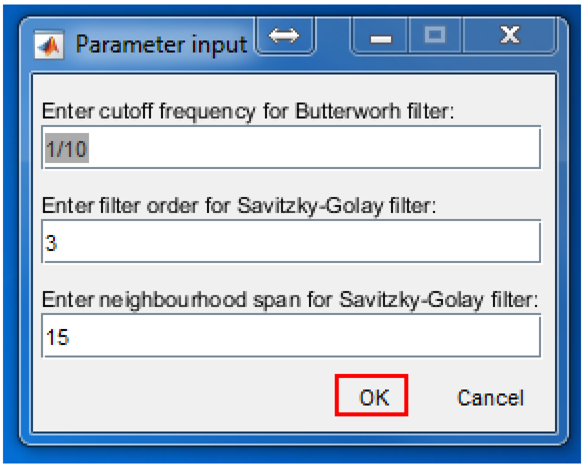

Fig. 1

3. In the next window that appears, select the tissue Doppler file to analyse and click 'Open'.

4. The automated software will automatically attempt to read the velocity scale, however, should this fail, you will be prompted to provide calibration.

5. Your image should appear on the screen, for you to enter the calibration. Select two points on the vertical (distance) scale, and type in the distance between them in cm (Fig. 2)

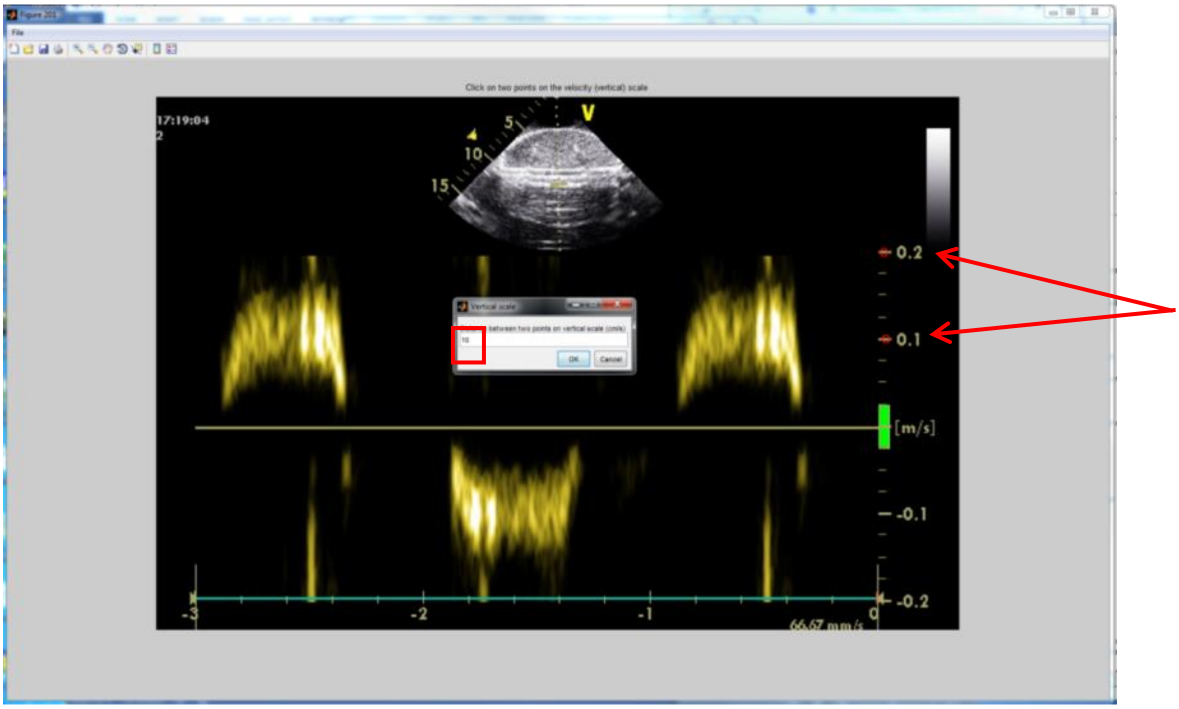

Fig. 2

6. Then select 2 points on the horizontal (time) scale, and type in the distance between them in seconds.

7. Finally, select the region of the Doppler image containing the trace (Fig. 3): green box):

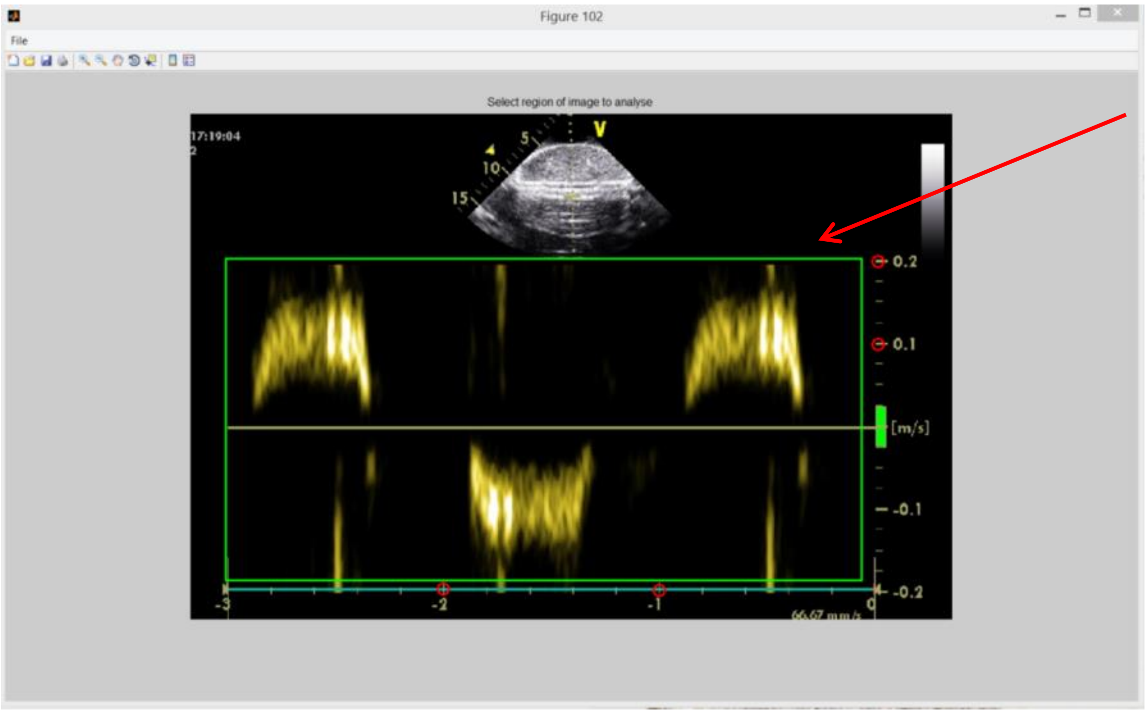

Fig. 3

8. Once the analysis is completed, you will see the automated trace overlaid on the original image (Fig. 4: red -outer line, blue -middle line):

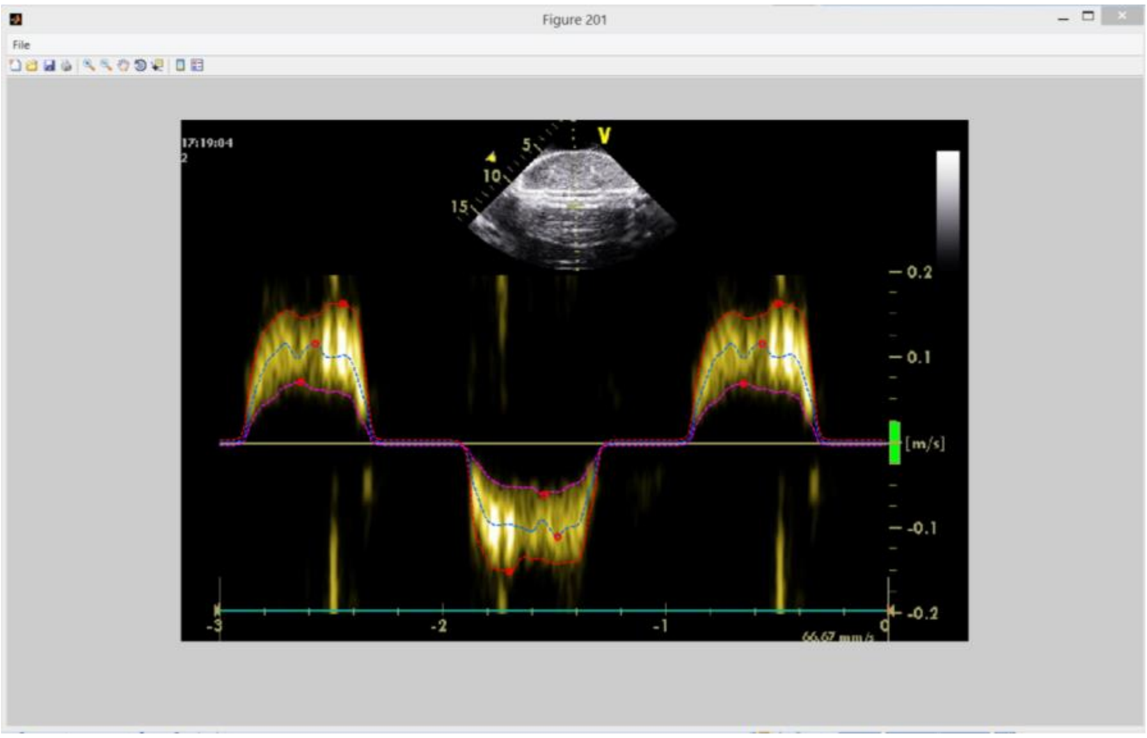

Fig. 4

9. The trace file and peak s′, e′ and a′ velocities measured automatically are saved in the same folder as the image, in a sub-folder 'TDI results'.

   Within the 'TDI results' folder you will find the following files:

   - '#name#-velocity_trace.bmp' is a snapshot of the automated traces overlaid on the image
   - 'TDI_vel_values.xls' contains automatic peak velocities
